# ﻿Two new phyllospheric species of *Colacogloea* (Colacogloeaceae, Pucciniomycotina) identified in China

**DOI:** 10.3897/mycokeys.101.114872

**Published:** 2024-01-12

**Authors:** Yun-Feng Lu, Chun-Yue Chai, Feng-Li Hui

**Affiliations:** 1 School of Life Science and Agricultural Engineering, Nanyang Normal University, Nanyang 473061, China Nanyang Normal University Nanyang China; 2 Research Center of Henan Provincial Agricultural Biomass Resource Engineering and Technology, Nanyang Normal University, Nanyang 473061, China Nanyang Normal University Nanyang China

**Keywords:** Basidiomycota, Microbotryomycetes, phyllosphere, phylogenetic analysis, taxonomy

## Abstract

During our ongoing survey of basidiomycetous yeasts associated with plant leaves in virgin forest, five *Colacogloea* strains were isolated in the Baotianman Nature Reserve, Henan Province, central China. Phenotypes from cultures and a phylogeny based on the internal transcribed spacer (ITS) regions and the D1/D2 domains of the large subunit (LSU) rRNA gene were employed to characterize and identify these isolates. As a result, two new species, namely *Colacogloeaceltidis***sp. nov.** and *C.pararetinophila***sp. nov.**, are introduced herein. In the phylogeny of combined ITS and LSU dataset, the new species *C.celtidis***sp. nov.** formed a clade with the unpublished *Colacogloea* strain (KBP: Y-6832), and together these formed the sister group to *C.armeniacae*, while *C.pararetinophila***sp. nov.** was retrieved as a sister to *C.retinophila*. A detailed description and illustration of both new species, as well as the differences between them and their closest relatives in the genus are provided. Results from the present study will add to our knowledge of the biodiversity of *Colacogloea* in China.

## ﻿Introduction

The genus *Colacogloea* consists of relatively rare and under-sampled dimorphic basidiomycetes (Sampaio et al. 2011). It was first proposed by Oberwinkler et al. (1990) to accommodate a single species, *C.effusa* (synonyms: *C.peniophorae*) which was initially described as *Platygloeaeffusa* (Bourdot and Galzin 1909). The genus *Colacogloea* was later expanded by the inclusion of *C.bispora* (originally described as *Platygloeabispora*) as a new combination (comb. nov.) (Oberwinkler et al. 1999), as well as two new species, *C.papilionacea* and *C.allantospora*, proposed by Kirschner and Oberwinkler (2000) and Bandoni et al. (2002), respectively. In 2015, Wang et al. revised the genus *Colacogloea* based on a multi-gene phylogeny, and transferred eight asexual species to the genus as the new combinations, *C.cycloclastica*, *C.diffluens*, *C.eucalyptica*, *C.falcata*, *C.foliorum*, *C.philyla*, *C.retinophila*, and *C.terpenoidalis* (Wang et al. 2015a, 2015b). Since then, six new members of the genus, *C.subericola* (originally described as *Rhodotorulasubericola*) from Spain (Belloch et al. 2007; Li et al. 2020), *C.demeterae* from Germany (Yurkov et al. 2016), and *C.aletridis*, *C.hydrangeae*, *C.armeniacae* and *C.rhododendri* from China (Li et al. 2020; Wang et al. 2021), were introduced based on morphological analyses and phylogenetic data. Recent incorporations into the genus are *C.bettinae*, *C.biconidiata*, *C.fennica*, *C.microspora*, and *C.universitatis*-*gandavensis* isolated from the hymenium of *Peniophorellapubera* and *P.praetermissa* (Schoutteten et al. 2023). The genus *Colacogloea* was included in the newly proposed family Colacogloeaceae within Microbotryomycetes (Wang et al. 2015b).

Until now, 23 species have been accepted in the genus *Colacogloea* (Wang et al. 2021; Schoutteten et al. 2023). Among them, *C.allantospora*, *C.bettinae*, *C.biconidiata*, *C.bispora*, *C.effusa*, *C.fennica*, *C.microspora*, *C.papilionacea*, *C.philyla*, and *C.universitatis*-*gandavensis* are known from their sexual states, which mostly developed in the fructifications of Polyporales, with transversely septate basidia, “simple” septal pores, and colacosomes (Oberwinkler et al. 1990; Sampaio et al. 2011; Schoutteten et al. 2023). The other 13 species are asexual morphs that resemble yeasts from the genus *Rhodotorula* and reproduce by polar budding (Hamamoto et al. 2011; Sampaio 2011; Wang et al. 2015b). Physiologically, the members of the genus *Colacogloea* all lack fermentative ability, possess Q-10 as a predominant ubiquinone, and assimilate various carbon sources, but not myo-inositol and methanol (Hamamoto et al. 2011; Sampaio 2011; Sampaio et al. 2011; Wang et al. 2015b).

The Baotianman Nature Reserve, located in Henan Province, Central China, measures 4,285 ha. With a forest coverage rate of 98%, it is classified as World Biosphere Reserve by the United Nations Educational, Scientific and Cultural Organization (UNESCO). The reserve encompasses a virgin forest with more than 2000 species of vascular plant. The local climate is typical of a transitional climate from northern subtropical zone to warm temperate zone, with cold dry winters, and fresh rainy summers, and an annual mean temperature of 15 °C (Hu et al. 2022). These weather patterns make Baotianman an excellent location for studying fungal diversity. During the survey, we collected several yeast strains of interest and used morphological comparison together with phylogenetic analyses to determine their classifications. As a result, we identified and characterized two new species of *Colacogloea*.

## ﻿Materials and methods

### ﻿Sample collection and yeast isolation

Leaf samples collected from Baotianman Nature Reserve were stored in sterile flasks and transported to the laboratory within 24 h. Yeast strains were isolated from leaf surfaces by the improved ballistospore-fall method as described in previous papers (Nakase and Takashima 1993; Hu et al. 2022). Vaseline was used to affix the semi-withered leaves onto the insides of Petri dishes filled with yeast extract-malt extract (YM) agar (0.3% yeast extract, 0.3% malt extract, 0.5% peptone, 1% glucose, and 2% agar). The dishes were then incubated at 20 °C until visible colonies had formed. Different yeast morphotypes were selected from these colonies and purified by streaking on separate YM agar plates. After purification, yeast strains were suspended in YM broth supplemented with 20% (v/v) glycerol and stored at −80 °C. Cultures of all obtained isolates were preserved at the Microbiology Lab, Nanyang Normal University, Henan, China. All isolates used in this study and their origins are presented in Table 1.

**Table 1.** Yeast strains and isolation sources investigated in this study.

	Strain	Source	Location
*Colacogloeaceltidis*	NYUN 2210184^T^	Leaf of *Celtisbungeana*	Getiaopa, Baotianman Nature Reserve, Neixiang, Henan Province, China
	NYUN 221136	Undetermined leaf	Mayigou, Baotianman Nature Reserve, Neixiang, Henan Province, China
*Colacogloeapararetinophila*	NYNU 2110393^T^	Undetermined leaf	Mayigou, Baotianman Nature Reserve, Neixiang, Henan Province, China
	NYNU 2110421	Undetermined leaf	Tianmanpubu, Baotianman Nature Reserve, Neixiang, Henan Province, China
	NYNU 2211185	Undetermined leaf	Tianmanpubu, Baotianman Nature Reserve, Neixiang, Henan Province, China

### ﻿Morphological and physiological characterization

Morphological and physiological characteristics of yeast strains were defined according to methods established by Kurtzman et al. (2011). Colony characteristics were observed and recorded on YM agar after two weeks of incubation at 20 °C. To investigate mycelium formation, colonies were transferred to corn meal (CM) agar (2% cornmeal infusion and 2% agar) slide cultures and incubated at 20 °C for two weeks. Tests of sexual reproductive potential were conducted for individual strains and strain pairs on potato dextrose agar (PDA) (20% potato infusion, 2% glucose, and 1.5% agar), CM agar, and yeast carbon base plus 0.01% ammonium sulphate (YCBS) agar for two months and observed at weekly intervals (Sampaio et al. 2011; Li et al. 2020). The inverted-plate method (do Carmo-Sousa and Phaff 1962) was used to observe the ballistoconidium-forming activity of all yeasts after two weeks of incubation on CM agar at 20 °C. Glucose fermentation was carried out in liquid medium using Durham fermentation tubes. Carbon and nitrogen source assimilation tests were conducted in liquid medium and starved inoculum was used for the nitrogen test (Kurtzman et al. 2011). Cycloheximide resistance was performed in liquid medium, while urea hydrolysis was conducted on agar slants. Acid production and diazonium blue B (DBB) reactions were investigated using Petri dishes with solid medium. Growth at different temperatures (15, 20, 25, 30, 35, and 37 °C) was determined by the amount of cultivation on YM agar. Cell morphology was examined using a Leica DM 2500 microscope (Leica Microsystems GmbH, Wetzlar, Germany) and a Leica DFC295 digital microscope color camera under bright field, phase contrast, or differential interference contrast (DIC) environment. All novel taxonomic descriptions and proposed names were deposited in the MycoBank database (http://www.mycobank.org; 29 October 2023).

### ﻿DNA extraction, PCR amplification, and sequencing

The total genomic DNA was extracted from yeast strains using the Ezup Column Yeast Genomic DNA Purification Kit according to the manufacturer’s instructions (Sangon Biotech Co., Shanghai, China). Two nuclear loci, which include the ITS regions and the D1/D2 domains of the LSU rRNA gene, were amplified using ITS1/ITS4 (White et al. 1990) and NL1/NL4 (Kurtzman and Robnett 1998) primers, respectively. The amplifications were performed in a 25 µL reaction-volume tube containing 9.5 µL of ddH_2_O, 12.5 µL of 2 × Taq PCR Master Mix with blue dye (Sangon Biotech Co., Shanghai, China), 1 µL of DNA template, and 1 µL of each primer. The following parameters were used to amplify the ITS and D1/D2 regions: an initial denaturation step of 2 min at 95 °C, followed by 35 cycles of 30 s at 95 °C, 30 s at 51 °C, 40 s at 72 °C, and a final extension of 10 min at 72 °C (Wang et al. 2014). The PCR products were purified and sequenced at Sangon Biotech Co., Ltd (Shanghai, China) with the same primers. We determined the identity and accuracy of the newly-obtained sequences by comparing them to sequences in GenBank and assembled them using BioEdit 7.1.3.0 (Hall 1999). All newly generated sequences were deposited in the GenBank database (https://www.ncbi.nlm.nih.gov/genbank/), and the accession numbers are listed in Table 2.

**Table 2.** Taxon names, strain numbers, and GenBank accession numbers used for phylogenetic analyses. Entries in bold were newly generated for this study.

Species Name	Strain No.	GenBank Accession No	
		ITS	LSU D1/D2
*Colacogloeaaletridis*	CBS 15459^T^	NR_174802	MK050450
*Colacogloeaarmeniacae*	CGMCC 2.6134^T^	MT252007	MT252007
*Colacogloeabettinae*	DSM 112418^T^	OQ870173	OQ875008
*Colacogloeabiconidiata*	DSM 112405^T^	OQ870175	OQ875010
***Colacogloeaceltidis***	**NYUN 2210184** ^T^	**OP954665**	**OP954664**
***Colacogloeaceltidis***	**NYUN 221136**	**OR727350**	**OR727349**
*Colacogloeacycloclastica*	CBS 8448^T^	NR_154750	NG_058729
*Colacogloeademeterae*	CBS 12500^T^	—	FN428967
*Colacogloeadiffluens*	CBS 5233^T^	NR_073289	NG_058991
*Colacogloeaeffusa*	DSM 113583^ET^	OQ870184	OQ875017
*Colacogloeaeucalyptica*	CBS 8499^T^	NR_111685	NG_058758
*Colacogloeafalcata*	CBS 7368^T^	NR_073297	NG_058723
*Colacogloeafennica*	DSM 113583^ET^	OQ870184	OQ875017
*Colacogloeafoliorum*	CBS 5234^T^	NR_073331	NG_058992
*Colacogloeahydrangeae*	CBS 15463^T^	NR_174803	MK050451
*Colacogloeamicrospora*	DSM 112413^T^	OQ870193	OQ875026
*Colacogloeapapilionacea*	RoKi 618^T^	—	EF450545
***Colacogloeapararetinophila***	**NYNU 2110393** ^T^	**OM014194**	**OM014193**
***Colacogloeapararetinophila***	**NYNU 2110421**	**OR727348**	**OR727347**
***Colacogloeapararetinophila***	**NYNU 2211185**	**OR727352**	**OR727351**
*Colacogloeaphilyla*	CBS 6272^T^	NR_073274	NG_058993
*Colacogloearetinophila*	CBS 8446^T^	NR_154830	NG_058994
*Colacogloearhododendri*	CBS 15652^T^	NR_174804	MK050452
*Colacogloeasubericola*	CBS 10442^T^	NR_137680	NG_060065
*Colacogloeaterpenoidalis*	CBS 8445^T^	NR_154749	NG_058995
*Colacogloeauniversitatis-gandavensis*	NS 20-022P^T^	—	OQ875007
*Colacogloea* sp.	KBP: Y-6832	ON263266	ON263266
*Chrysozymagriseoflava*	CBS 7284^T^	NR_073303	NG_058746
*Udeniozymaferulica*	CBS 7416^T^	NR_073330	NG_058429
*Yurkovialongicylindrica*	CGMCC 2.5603^T^	NR_174799	MK050441

CBS, CBS-KNAW Collections, Westerdijk Fungal Biodiversity Institute, Utrecht, The Netherlands; DSM, German Collection of Microorganisms and Cell Cultures GmbH, Braunschweig, Germany; CGMCC, China General Microbiological Culture Collection Center, Beijing, China; NYNU, Microbiology Lab, Nanyang Normal University, Henan, China; T, ex-type strain; ET, ex-epitype strain. Species obtained in this study are in bold.

### ﻿Phylogenetic analysis

A total of 57 nucleotide sequences that belonged to 27 taxa were included in the phylogenetic analyses. Except for 10 sequences recognized in this study, the other sequences were obtained from previous studies (Li et al. 2020; Wang et al. 2021) and GenBank (Table 2). *Udeniozymaferulica*CBS 7416^T^ was used as the outgroup. The phylogenetic relationships of the new *Colacogloea* species and their relatives were determined using a combined ITS and LSU sequence dataset. Sequences of the individual loci were aligned with Clustal X 1.83 (Thompson et al. 1997) or MAFFT 7.110 (Katoh and Standley 2013) using default settings. PhyloSuite V1.2.2 (Zhang et al. 2020) was used to concatenate the aligned sequences of the different loci. The few ambiguously aligned regions of the ITS and LSU alignments were removed with Gblocks v.0.91b, by keeping the default settings but allowing all gap positions when not ambiguous and manually adjusted in Sequencher 5.4.5 (Katoh et al. 2019; Castresana 2000).

Phylogenetic analyses were carried out using maximum likelihood (ML) and Bayesian inference (BI) methods. The ML analysis was conducted with RAxML v. 8.2.3 (Stamatakis 2014) using a GTRGAMMA substitution model. ML bootstrap values (MLBS) of the nodes were evaluated using 1,000 rapid bootstrap replicates. For the BI approach, ModelFinder (Kalyaanamoorthy et al. 2017) was used to determine the appropriate substitution model that would best fit the DNA evolution for the combined dataset. MrBayes 3.2.7a (Ronquist et al. 2012) in the CIPRES Science Gateway version 3.3 was used to analyze the BI data. Best-fit evolution models were determined as GTR+I+G for the ITS and LSU partitions. Six simultaneous Markov chains were run for 50 million generations and trees were sampled every 1,000^th^ generation. The first 25% of created sample trees were discarded as they represent the burn-in phase of analysis. The remaining trees were used to calculate the Bayesian posterior probabilities (BPP) of the clades.

The resulting trees were viewed in FigTree v. 1.4.3 (Andrew 2016) and processed with Adobe Illustrator CS5. Branches that received MLBS ≥ 50% and BPP ≥ 0.95 were considered significantly supported.

## ﻿Results

### ﻿Phylogenetic analysis

**Figure 1.**
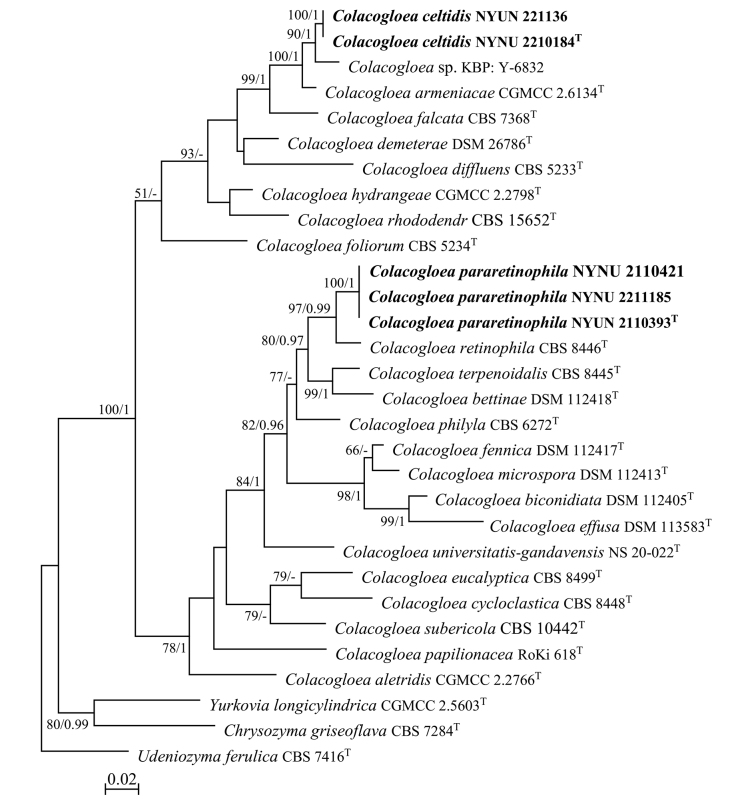
Maximum likelihood (ML) phylogram of *Colacogloea* species based on combined ITS and LSU sequence data. *Udeniozymaferulica*CBS 7416^T^ was used as the outgroup. Branches are labeled with MLBS ≥ 50% and BPP ≥ 0.95. New strains described in this study are shown in bold.

During this study, five strains of *Colacogloea* were discovered in the Baotianman Nature Reserve. To reveal the phylogenetic position of the specimens, we performed phylogenetic analyses with combined ITS and LSU sequence data. The dataset consisted of 1,272 characters (674 characters from ITS and 598 characters from LSU), of which 715 were constant, 536 were variable, 366 were parsimony-informative, and 164 were singleton. ML and BI analyses generated similar topologies, with the BI analysis reaching an average standard deviation of split frequencies of 0.009922. The consensus topology from the ML analysis with MLBS (≥ 50%) and BPP (≥ 0.95) labeled on branches is shown (Fig. 1). In the phylogenetic trees, five strains isolated in this study formed two strongly supported groups (100% MLBS/1 BPP), and were clearly distinct from other known species of *Colacogloea*.

The two strains NYUN 2210184^T^ and NYUN 221136 possess identical sequences in both the D1/D2 domains and ITS regions, indicating they belong to same species. The NYUN 2210184^T^ group formed a well-supported clade and then grouped with the unpublished strain *Colacogloea* sp. KBP: Y-6832 and *C.armeniacae*, with strong support (100 MLBS/1 BPP; Fig. 1). The D1/D2 sequences of this group differed by only 3 nt substitutions (~0.5%) from *Colacogloea* sp. KBP: Y-6832; however, there were 16 nt (~2.9%) differences in the ITS regions, which indicates that the isolate KBP: Y-6832 may represent a different species. Similarly, the NYUN 2210184^T^ group differed from the type strain of the closest known species *C.armeniacae* by 6 nt (~1%) substitutions in the D1/D2 domains and by more than 15 nt (~2.5%) mismatches in the ITS regions. According to the basidiomycetous yeast species thresholds proposed by Fell et al. (2000), Scorzetti et al. (2002), and Vu et al. (2016), strains that differ by two or more nucleotide substitutions in the D1/D2 domains or 1–2% nucleotide differences in the ITS regions may represent different taxa. Therefore, the differences in both the D1/D2 and ITS sequences were significant enough for the NYUN 2210184^T^ group to be considered a distinct *Colacogloea* species.

Three strains NYNU 2110393^T^, NYNU 2110421, and 2211185 formed a well-supported clade (100% MLBS/1 BPP; Fig. 1). They shared a 100% of nucleotide identity based on their D1/D2 and ITS sequences, indicating that they are conspecific. The closest relative of the NYNU 2110393^T^ group is *C.retinophila*, but differed from the type strain of the latter by six nt (~1%) substitutions in the D1/D2 domains and 31 nt (~5%) mismatches in the ITS regions, respectively. According to the criteria mentioned above, this data clearly supports the distinction between the NYNU 2110393^T^ group and *C.retinophila* at the species level.

## ﻿Taxonomy

### ﻿ Colacogloea celtidis

Taxon classificationFungiHeterogastridialesColacogloeaceae

C.Y. Chai & F.L. Hui sp. nov.

7FF46A19-8FD6-5F62-BDCA-4672F50D0AD0

850696

Fig. 2A

#### Etymology.

The specific epithet “*celtidis*” refers to *Celtis*, the plant genus, from which the type strain was isolated.

#### Typus.

China, Henan Province, Neixiang County, Baotianman Nature Reserve, Getiaopa (33°29′07″N, 111°52′51″E), in phylloplane from leaf of *Celtisbungeana*, October 2022, J.Z. Li, NYUN 2210184 (holotype GDMCC 2.332^T^ preserved as a metabolically inactive state, culture ex-type KCTC 37265 and CICC 33577).

#### Description.

On YM agar, after two weeks at 20 °C, the streak culture is cream, butyrous, and smooth. The margin is entire. In YM broth, after 7 d at 20 °C, cells are long cylindrical, 2.3–3.0 × 7.0–10.2 μm and single, budding is polar. After 1 mo at 20 °C, a ring and sediment are present. In Dalmau plate culture on corn meal agar, hyphae and pseudohyphae are not formed. Sexual structures are not observed for individual strains and strain pairs on PDA, CM agar and YCBS agar for two months. Ballistoconidia are not produced. Glucose fermentation is absent. Glucose, salicin, D-xylose (weak), D-arabinose, 5-keto-D-gluconate, ethanol (weak), glycerol, ribitol, D-mannitol, D-glucitol, succinate, D-gluconate, D-glucosamine (weak), 2-keto-D-gluconate, D-glucuronate, and glucono-1,5-lactone are assimilated as sole carbon sources. Inulin, sucrose, raffinose, melibiose, galactose, lactose, trehalose, maltose, melezitose, methyl-α-D-glucoside, cellobiose, L-sorbose, L-rhamnose, L-arabinose, D-ribose, methanol, erythritol, galactitol, myo-inositol, DL-lactate, and citrate are not assimilated. Nitrate, nitrite (delayed and weak), ethylamine (delayed and weak), and L-lysine (delayed) are assimilated as sole nitrogen sources. Cadaverine is not assimilated. Maximum growth temperature is 30 °C. Growth in vitamin-free medium is positive. Starch-like substances are not produced. Urease activity is positive. Diazonium Blue B reaction is positive.

**Figure 2.**
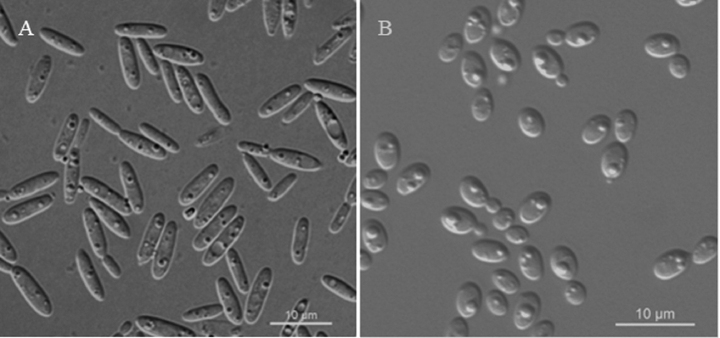
Vegetative cells of *Colacogloeaceltidis* sp. nov. NYUN 2210184^T^ (**A**) and *Colacogloeapararetinophila* sp. nov. NYNU 2110393^T^ (**B**) following growth in YM broth for 7 days at 20 °C. Scale bars: 10 μm.

#### Additional strain examined.

China, Henan Province, Neixiang County, Baotianman Nature Reserve, Mayigou (33°30′44″N, 111°55′47″E) in phylloplane from undetermined leaf, October 2022, J.Z. Li, NYNU 221136.

#### GenBank accession numbers.

Holotype NYUN 2210184^T^ (ITS: OP954665, D1/D2: OP954664); additional strain NYUN 221136 (ITS: OR727350, D1/D2: OR727349).

#### Note.

Phylogenetic analyses revealed that *C.celtidis* sp. nov. formed a single clade with high support (100 MLBP/1 BPP; Fig. 1). *C.eltise* sp. nov. can be physiologically differentiated from its closest known species *C.armeniacae* (Wang et al. 2021) by its ability to grow in D-xylose and D-arabinose and inability to grow in trehalose and cellobiose (Table 3).

**Table 3.** Physiological and biochemical characteristics that differ between the new species and closely related species.

Characteristics	*C.celtidis*	C.armeniacae*	*C.pararetinophila*	C.retinophila*
Carbon assimilation				
Trehalose	–	+	w	+
Cellobiose	–	+	–	–
Salicin	+	–/d/w	w	–
D-Xylose	w	–	+	s
D-Arabinose	+	–	+	s
D-Ribose	–	–/d/w	+	–
D-Glucuronate	+	+	–	+
Nitrogen assimilation				
Nitrate	+	+	+	–
Nitrite	d/w	+	+	–
L-Lysine	d	–	+	n
Growth tests				
0.1% Cycloheximide	–	n	+	–

+, positive reaction; –, negative reaction; d, delayed positive; s, slowly positive; w, weakly positive; n, data not available. All data from this study, except* which were obtained from the original description (Sampaio 2011; Wang et al. 2021).

### ﻿ Colacogloea pararetinophila

Taxon classificationFungiHeterogastridialesColacogloeaceae

C.Y. Chai & F.L. Hui sp. nov.

7A8D445E-AF1A-5C0E-8B4C-A84AACE408CF

850697

Fig. 2B

#### Etymology.

The specific epithet “*pararetinophila*” refers to its phylogenetic similarity to *C.retinophila*.

#### Typus.

China, Henan Province, Neixiang County, Baotianman Nature Reserve, Mayigou (33°30′44″N, 111°55′47″E) in phylloplane from undetermined leaf, October 2021, W.T. Hu and R.Z. Qiao, NYNU 2110393 (holotype CICC 33533^T^ preserved as a metabolically inactive state, culture ex-type JCM 35724 and GDMCC 2.268).

#### Description.

On YM agar, after two weeks at 20 °C, the streak culture is cream, butyrous, and smooth. The margin is entire. In YM broth, after 7 d at 20 °C, cells are ovoid or ellipsoidal, 2.0–2.6 × 2.8–4.2 μm and single, budding is polar. After 1 mo at 20 °C, a ring and sediment are present. In Dalmau plate culture on corn meal agar, hyphae and pseudohyphae are not formed. Sexual structures are not observed for individual strains and strain pairs on PDA, CM agar and YCBS agar for two months. Ballistoconidia are not produced. Glucose fermentation is absent. Glucose, trehalose (weak), salicin (weak), D-xylose, D-arabinose, 5-keto-D-gluconate, D-ribose, ethanol, glycerol, ribitol, D-mannitol, D-glucitol, succinate (weak), citrate (weak), D-gluconate, D-glucosamine, 2-keto-D-gluconateare, and glucono-1,5-lactone assimilated as sole carbon sources. Inulin, sucrose, raffinose, melibiose, galactose, lactose, maltose, melezitose, methyl-α-D-glucoside, cellobiose, L-sorbose, L-rhamnose, L-arabinose, methanol, erythritol, galactitol, myo-inositol, DL-lactate, and D-glucuronate are not assimilated. Nitrate, nitrite, ethylamine, and L-lysine are assimilated as sole nitrogen sources. Cadaverine is not assimilated. Maximum growth temperature is 37 °C. Growth in vitamin-free medium is positive. Starch-like substances are not produced. Urease activity is positive. Diazonium Blue B reaction is positive.

#### Additional strain examined.

China, Henan Province, Neixiang County, Baotianman Nature Reserve, Tianmanpubu (33°30′26″N, 112°02′28″E) in phylloplane from undetermined leaf, October 2021, W.T. Hu and R.Z. Qiao, NYNU 2110421 and October 2022, J.Z. Li, NYUN 2211185.

#### GenBank accession numbers.

Holotype NYNU 2110393^T^ (ITS: OM014194, D1/D2: OM014193); additional strains NYNU 2110421 (ITS: OR727348, D1/D2: OR727347) and NYUN 2211185 (ITS: OR727352, D1/D2: OR727351).

#### Note.

Phylogenetic analyses revealed that *C.pararetinophila* sp. nov. has a close relationship with *C.retinophila* with high support values (100 MLBP/1 BPP; Fig. 1). *C.pararetinophila* sp. nov. can be physiologically differed from its closest relative *C.retinophila* (Sampaio 2011) in the ability to assimilate salicin and D-ribose and inability to assimilate D-glucuronate. In addition, *C.pararetinophila* sp. nov. can grow in 0.1% cycloheximide while *C.retinophila* cannot (Table 3).

## ﻿Discussion

Traditional methods of classification for *Colacogloea* species are based primarily on phenotypical features, such as colony morphology, cell shape, basidia formation, details of physiological and biochemical characteristics, etc. (Sampaio et al. 2011). The classification based on these phenotypical features, however, was in many cases not consistent with the results obtained from phylogenetic analyses. For example, *R.cycloclastica*, *R.philyla*, and *R.retinophila*, originally classified in the polyphyletic anamorphic genus *Rhodotorula*, are nested within the genus *Colacogloea* based on phylogenetic analyses (Sampaio et al. 2011; Wang et al. 2015a). As a result, these three species were then reassigned to the genus *Colacogloea*, according to the International Code of Nomenclature for Algae, Fungi, and Plants (McNeill et al. 2012). Therefore, a combination of phenotypical characteristics and phylogenetic analysis has been adopted as the standard method for concretely identifying *Colacogloea* species (Wang et al. 2015b).

In this study, we introduce *C.celtidis* sp. nov. and *C.pararetinophila* sp. nov. as two new species of *Colacogloea*, and describe them in asexual morphs based on molecular analyses and morphological features. Our phylogenetic analyses indicated that the genus of *Colacogloea* has two subclades (Fig. 1), which are in concordance with previous studies (Wang et al. 2015a; Li et al. 2020; Wang et al. 2021; Schoutteten et al. 2023). *C.celtidis* sp. nov., with its sister species *C.armeniacae*, form a well-separated clade in subclade I, which is comprised of anamorphic species only. *C.pararetinophila* sp. nov., with its sister species *C.retinophila*, form a monophyletic lineage in subclade II, which includes eight teleomorphic species and seven anamorphic species. In previous studies, the two sub-clades were well supported in phylogenetic trees from the four protein-coding genes and the combined seven-loci analysis (Wang et al. 2015a; Wang et al. 2021; Schoutteten et al. 2023). Multi-gene phylogenetic analyses suggest that the two sub-clades of the genus *Colacogloea* seem to represent two genera, although that was not well supported in the phylogenetic tree produced by this study (Fig. 1). Therefore, further analyses using more molecular data or genomic data are needed to clarify the possible heterogeneity of the genus.

*Colacogloea* species are widely distributed and are found in different habitats. Filamentous morphs of *Colacogloea* species were mainly isolated from the hymenia of corticioid fungi, especially from the genera *Peniophorella* and *Tubulicrinis* (Sampaio et al. 2011; Schoutteten et al. 2023). The yeast morphs of *Colacogloea* species can be isolated from leaves, fruits, tree bark, plant residues, soil, insects, and tunnels (Belloch et al. 2007; Hamamoto et al. 2011; Sampaio 2011; Sampaio et al. 2011; Yurkov et al. 2016; Li et al. 2020; Wang et al. 2021), but most of them are found mostly in association with plant materials, especially leaves. Moreover, the yeast morphs of *C.papilionacea* and *C.philyla*, that were isolated from insects and insect tunnels, were also collected from plants (Kirschner and Oberwinkler 2000; Sampaio 2011; Sampaio et al. 2011). In this study, the five isolates of two new species also have an association with plant leaves, like most of the other anamorphic species in the genus. Taken together, these findings might indicate that the plant is a common habitat of *Colacogloea* species in the yeast morphs.

## Supplementary Material

XML Treatment for Colacogloea celtidis

XML Treatment for Colacogloea pararetinophila

## ﻿Additional information

### Author ORCIDs

Yun-Feng Lu https://orcid.org/0000-0003-0284-5560

Chun-Yue Chai https://orcid.org/0000-0001-7753-6223

Feng-Li Hui https://orcid.org/0000-0001-7928-3055

### Data availability

All of the data that support the findings of this study are available in the main text or Supplementary Information.

## Supplementary materials

Supplementary material 1 Molecular data Data type fasta Explanation note A dataset of ITS and LSU for Fig. 1. https://binary.pensoft.net/file/965870This dataset is made available under the Open Database License (http://opendatacommons.org/licenses/odbl/1.0/). The Open Database License (ODbL) is a license agreement intended to allow users to freely share, modify, and use this Dataset while maintaining this same freedom for others, provided that the original source and author(s) are credited.Yun-Feng Lu, Chun-Yue Chai, Feng-Li Hui
